# Six‐Month Benralizumab Maintenance for Relapsing Chronic Eosinophilic Pneumonia Guided by Eosinophil Kinetics

**DOI:** 10.1002/rcr2.70515

**Published:** 2026-02-16

**Authors:** Toshiyuki Sumi, Taiki Ishigooka, Kazuya Takeda, Taeka Naraoka, Naoki Shijubou, Yuichi Yamada, Hirofumi Chiba

**Affiliations:** ^1^ Department of Pulmonary Medicine Hakodate Goryoukaku Hospital Hakodate Japan; ^2^ Department of Respiratory Medicine and Allergology Sapporo Medical University School of Medicine Sapporo Japan; ^3^ Department of Respiratory Medicine Asahikawa Medical University Hospital Asahikawa Japan

**Keywords:** benralizumab, chronic eosinophilic pneumonia, deep depletion, eosinophil kinetics, mepolizumab

## Abstract

Idiopathic chronic eosinophilic pneumonia (ICEP) often relapses upon corticosteroid tapering. Biologics targeting interleukin‐5 (IL‐5) are effective, but optimal dosing intervals remain unclear. We report a case of relapsing ICEP in a patient in her 50s. Mepolizumab, an IL‐5 ligand blocker, failed to maintain remission, with clinical relapse occurring 4 months after initiation. Switching to benralizumab, an interleukin‐5 receptor blocker, induced rapid and deep eosinophil depletion. For optimising dosing, treatment was temporarily withheld, revealing a prolonged remission duration of 8 months before eosinophil recovery and clinical relapse. Based on these kinetics, a 6‐monthly benralizumab maintenance strategy was established. The patient has remained relapse‐free with zero eosinophils for over 2 years under this biannual regimen. This case suggests that benralizumab offers superior durability compared to mepolizumab in CEP due to deep depletion, enabling an extended, cost‐effective dosing interval guided by individual eosinophil recovery kinetics.

## Graphical abstract

We report a case of relapsing chronic eosinophilic pneumonia where benralizumab showed superior durability compared to mepolizumab. Based on the eosinophil recovery kinetics (relapse at 8 months after withdrawal), a personalised maintenance strategy of benralizumab every 6 months was established, successfully maintaining remission for over 2 years.

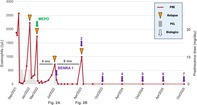

## 1 Introduction

Idiopathic chronic eosinophilic pneumonia (ICEP) is a rare disorder characterised by severe eosinophilic pulmonary infiltration. Although systemic corticosteroids are the standard treatment, relapses are frequent, occurring in more than 50% of patients during tapering or after cessation. Consequently, numerous patients require long‐term corticosteroid maintenance, which can lead to significant adverse effects. Recently, monoclonal antibodies targeting interleukin‐5 (IL‐5) (mepolizumab) or its receptor (benralizumab) have demonstrated efficacy in reducing relapses and sparing corticosteroids in eosinophilic diseases. However, the optimal dosing strategy for CEP—often based on asthma protocols (every 4 or 8 weeks)—remains to be established without fully considering the disease‐specific kinetics of eosinophil recovery. We herein report a case of relapsing CEP in which the durability of benralizumab was superior to that of mepolizumab, allowing for a personalised ‘every 6 months’ maintenance strategy guided by eosinophil kinetics.

## 2 Case Report

A female patient in her 50s presented with dyspnea and cough in September 2021. Although she had a history of bronchial asthma, it was in remission and did not require treatment with inhaled corticosteroids. She had no history of atopic dermatitis or smoking. Chest computed tomography (CT) revealed peripheral non‐segmental consolidations and ground‐glass opacities (GGOs). The peripheral blood eosinophil count was markedly elevated (1872 /μL). Comprehensive screening was performed to exclude secondary causes of eosinophilic pneumonia. Specific antibodies for parasites and stool examination were negative. Myeloperoxidase (MPO)‐ and proteinase 3 (PR3)‐anti‐neutrophil cytoplasmic antibodies (ANCA) were negative. There was no history of new drug exposure or overseas travel. Based on the characteristic radiographic findings and severe peripheral eosinophilia, and exclusion of other etiologies, the patient was clinically diagnosed with ICEP. Prednisolone (PSL) 30 mg/day was initiated, leading to rapid improvement. However, eosinophilia and symptoms recurred when the PSL dose was tapered to < 5 mg/day. Clinical features of hypereosinophilic syndrome (HES) with extra‐pulmonary organ involvement were absent.

**FIGURE 1:**
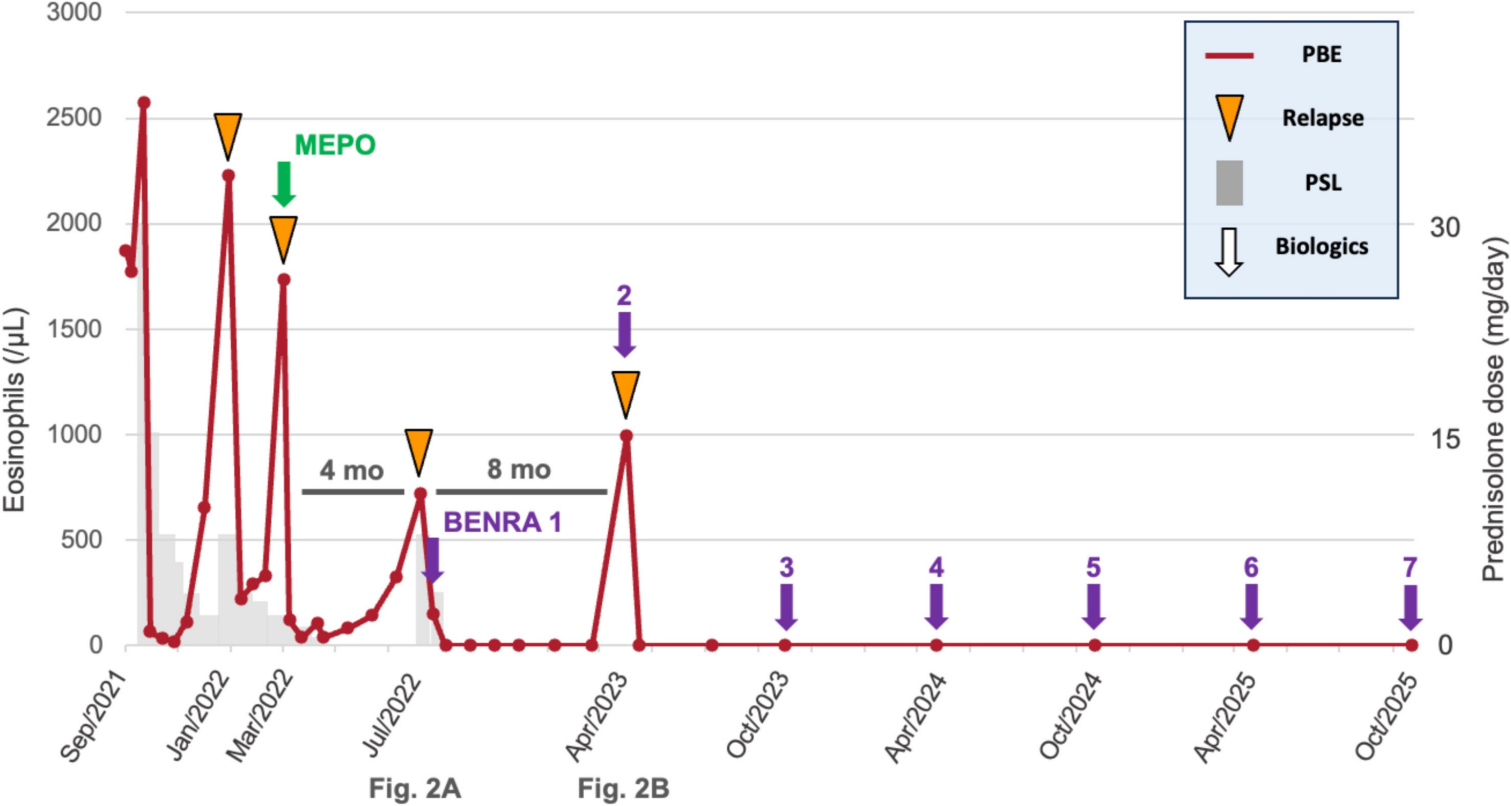
Clinical course showing eosinophil counts (red) and prednisolone dose (grey). Relapses occurred at 4 months (indicated as Figure 2A) and 8 months (indicated as Figure 2B) after treatment with mepolizumab and benralizumab, respectively. Since April 2023, benralizumab maintenance every 6 months (arrows) has led to sustained remission with no eosinophils for > 2 years.

**FIGURE 2:**
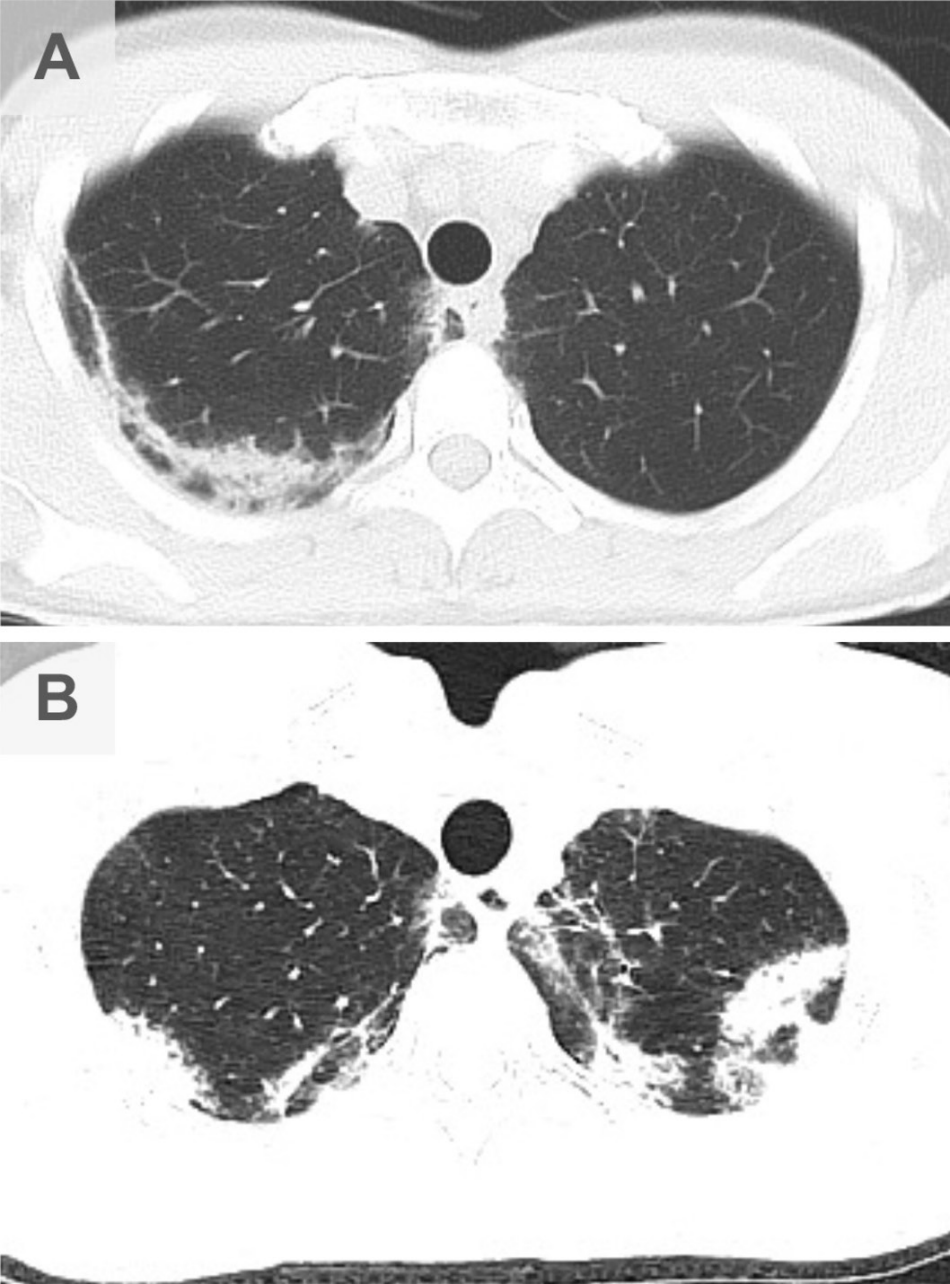
Chest computed tomography (CT) findings during clinical relapses (corresponding to time points A and B in Figure 1). (A) CT scan taken during relapse while on mepolizumab treatment (August 2022), showing non‐segmental ground‐glass opacities and consolidations predominantly in the right upper lobe. (B) CT scan taken during relapse 8 months after the last benralizumab dose (April 2023). Recurrence of bilateral multifocal consolidations and ground‐glass opacities indicates a return of active disease, similar to the previous flare.

In March 2022, she developed a second relapse due to steroid dependence (eosinophils 1737 /μL), and mepolizumab (100 mg SC) was introduced. Although the eosinophil count initially decreased, it began to re‐elevate in July 2022, approximately 4 months after initiation, accompanied by fever and worsening respiratory symptoms (Figure 1). Chest CT revealed the recurrence of GGOs and consolidation in the right upper lobe (Figure 2A). This suggested that mepolizumab was insufficient for maintaining remission.

The treatment was switched to benralizumab (30 mg SC) in August 2022, resulting in immediate and complete peripheral eosinophil depletion (0 /μL). Considering this rapid response, we sought to determine the duration of efficacy (eosinophil recovery time) for optimising the treatment interval. Benralizumab was temporarily withheld after the induction phase. The patient remained asymptomatic with zero eosinophils for more than half a year. A clinical relapse eventually occurred in April 2023, exactly 8 months after the last dose, coinciding with a rebound of eosinophils to 996 /μL. Chest CT revealed multifocal consolidations and GGOs, similar to the previous flares (Figure 2B).

Based on the observed kinetics, where relapse occurred at 8 months, we established a maintenance strategy of benralizumab every 6 months to prevent recurrence while minimising drug exposure. Under this biannual regimen, peripheral eosinophils remained consistently at 0 /μL. As of October 2025, the patient has been relapse‐free without oral corticosteroids for over 2 years (Figure 1).

## 3 Discussion

This case highlights two critical clinical insights: the superior durability of benralizumab over mepolizumab in CEP, and the feasibility of an extended dosing interval.

First, the variation in time‐to‐relapse (4 vs. 8 months) likely reflects distinct mechanisms of action. Mepolizumab binds to circulating IL‐5 and neutralises its effects. While it arrests eosinophil maturation in the bone marrow, it does not deplete eosinophil progenitors [1]. Brenard et al. reported that while mepolizumab reduced the relapse rates in real‐world patients with CEP, some patients showed residual radiographic opacities or an inability to wean off steroids completely [2]. In our case, the early relapse on mepolizumab suggests that the bone marrow ‘factory’ remained active, leading to rapid repopulation once antibody levels waned. In contrast, benralizumab targets the IL‐5 receptor α subunit and induces antibody‐dependent cell‐mediated cytotoxicity. This mechanism depletes mature eosinophils and bone marrow progenitors (‘deep depletion’) [3]. We hypothesised that, because benralizumab destroyed the progenitor pool, it took significantly longer (8 months) for the eosinophil lineage to recover. Recent data from the MANDARA trial on EGPA also support the non‐inferiority and potential superiority of benralizumab over mepolizumab in maintaining remission [4].

Second, this case supports the concept of ‘eosinophil kinetics‐guided’ dosing. While biomarker‐guided strategies are common for corticosteroid tapering, applying them to optimise biologic regimens is crucial but underutilised. Standard biological regimens are fixed, which may result in overtreatment for patients with CEP who achieve deep depletion. Unlike corticosteroids or mepolizumab, benralizumab's ‘deep depletion’ mechanism creates a prolonged recovery phase, enabling a significantly longer dosing interval. Izumo et al. previously reported a case of CEP in which a single dose of benralizumab maintained remission for approximately 6 months [5]. Although extending dosing intervals raises concerns about the development of anti‐drug antibodies (ADA), which has been reported to occur more frequently with less frequent dosing [6, 7], our patient has maintained clinical efficacy without secondary failure for over 2 years. Careful long‐term monitoring for ADA‐related loss of efficacy is warranted. Nevertheless, our 2‐year follow‐up confirms that a 6‐month interval is a reproducible and safe strategy for maintaining long‐term remission. This approach not only reduces the total drug exposure, medical costs and physical burden on the patient.

In conclusion, benralizumab provided superior disease control compared with mepolizumab in this patient with relapsing CEP. Optimising biological therapy based on individual eosinophil recovery kinetics is a promising precision‐medicine approach for CEP management.

## Author Contributions

T.S. managed the patient and wrote the manuscript. T.I., K.T., T.N., N.S., Y.Y. and H.C. assisted with the data collection and reviewed the manuscript. All authors read and approved the final manuscript.

## Funding

The authors have nothing to report.

## Consent

The authors declare that written informed consent was obtained for the publication of this manuscript and accompanying images and attest that the form used to obtain consent from the patient complies with the Journal requirements as outlined in the author guidelines.

## Conflicts of Interest

The authors declare no conflicts of interest.

## Data Availability

The data that support the findings of this study are available from the corresponding author upon reasonable request.

## References

[1] A. Menzies‐Gow , P. Flood‐Page , R. Sehmi , et al., “Anti‐IL‐5 (Mepolizumab) Therapy Induces Bone Marrow Eosinophil Maturational Arrest and Decreased Mobilization Into the Blood and Airways in Atopic Subjects,” Journal of Allergy and Clinical Immunology 111, no. 4 (2003): 714–719. 12704348 10.1067/mai.2003.1382

[2] E. Brenard , C. Pilette , C. Dahlqvist , et al., “Real‐Life Study of Mepolizumab in Idiopathic Chronic Eosinophilic Pneumonia,” Lung 198, no. 2 (2020): 355–360. 32052155 10.1007/s00408-020-00336-3

[3] M. Laviolette , D. L. Gossage , G. Gauvreau , et al., “Effects of Benralizumab on Airway Eosinophils in Asthmatic Patients With Sputum Eosinophilia,” Journal of Allergy and Clinical Immunology 132, no. 5 (2013): 1086–1096.e5. 23866823 10.1016/j.jaci.2013.05.020 PMC4172321

[4] M. E. Wechsler , P. Nair , B. Terrier , et al., “Benralizumab Versus Mepolizumab for Eosinophilic Granulomatosis With Polyangiitis,” New England Journal of Medicine 390, no. 8 (2024): 911–921. 38393328 10.1056/NEJMoa2311155

[5] T. Izumo , N. Kuse , N. Awano , et al., “Rapid and Sustained Effects of a Single Dose of Benralizumab on Chronic Eosinophilic Pneumonia,” Respiratory Medicine Case Reports 30 (2020): 101062. 32373456 10.1016/j.rmcr.2020.101062 PMC7193122

[6] E. L. Howard , M. M. Goens , L. Susta , A. Patel , and S. K. Wootton , “Anti‐Drug Antibody Response to Therapeutic Antibodies and Potential Mitigation Strategies,” Biomedicine 13, no. 2 (2025): 299. 10.3390/biomedicines13020299 PMC11853408 40002712

[7] M. L. Chen , T. Nopsopon , and A. Akenroye , “Incidence of Anti‐Drug Antibodies to Monoclonal Antibodies in Asthma: A Systematic Review and Meta‐Analysis,” Journal of Allergy and Clinical Immunology 11, no. 5 (2023): 1475–1484.e20. 10.1016/j.jaip.2022.12.046 PMC10601343 36716995

